# NeuroMem: Analog Graphene-Based Resistive Memory for Artificial Neural Networks

**DOI:** 10.1038/s41598-020-66413-y

**Published:** 2020-06-11

**Authors:** Heba Abunahla, Yasmin Halawani, Anas Alazzam, Baker Mohammad

**Affiliations:** 1 System on Chip Center, ECE, Khalifa University, Abu Dhabi, UAE; 2 System on Chip Center, MECH, Khalifa University, Abu Dhabi, UAE

**Keywords:** Electrical and electronic engineering, Electronic devices, Electronic properties and devices

## Abstract

Artificial Intelligence (AI) at the edge has become a hot subject of the recent technology-minded publications. The challenges related to IoT nodes gave rise to research on efficient hardware-based accelerators. In this context, analog memristor devices are crucial elements to efficiently perform the multiply-and-add (MAD) operations found in many AI algorithms. This is due to the ability of memristor devices to perform in-memory-computing (IMC) in a way that mimics the synapses in human brain. Here, we present a novel planar analog memristor, namely NeuroMem, that includes a partially reduced Graphene Oxide (prGO) thin film. The analog and non-volatile resistance switching of NeuroMem enable tuning it to any value within the R_ON_ and R_OFF_ range. These two features make NeuroMem a potential candidate for emerging IMC applications such as inference engine for AI systems. Moreover, the prGO thin film of the memristor is patterned on a flexible substrate of Cyclic Olefin Copolymer (COC) using standard microfabrication techniques. This provides new opportunities for simple, flexible, and cost-effective fabrication of solution-based Graphene-based memristors. In addition to providing detailed electrical characterization of the device, a crossbar of the technology has been fabricated to demonstrate its ability to implement IMC for MAD operations targeting fully connected layer of Artificial Neural Network. This work is the first to report on the great potential of this technology for AI inference application especially for edge devices.

## Introduction

Memristor device (MR), a resistor with a memory, is an emerging resistive random access memory (RRAM) type technology that plays a great role beyond Complementary Metal Oxide Semiconductor (CMOS)-based platforms with extendable performance. MR was firstly postulated by Leon Chua in 1971^1^. Although many researches have been carried out related memristive switching phenomena^2^, the first link to Chua’s theory was done by HP labs in 2008^3^. After this, the MR research started to gain an increasing attention, especially in device fabrication and optimization. One of the key features of MR device is its ability to work as an analog memory that mimics the synapse behavior in the brain and consequently achieve a bio-inspired system. MR devices can be fabricated with a synaptic density greater than that of human brain tissue^4,5^. Neuromorphic systems based on such circuits produce high density and extreme low-power hardware designs that are capable of performing many MAD operations in parallel in the analog domain^6–8^. Thus, efficient mapping of neural network algorithms can be translated to MR operations to achieve MR-based deep system^9^.

From this perspective, numerous recent works report on multi-state MR devices, as the gradual change in resistivity emulates the synapse plasticity change in the brain^10–16^. Many metal oxides (e.g. CuO, TiO_2_, HfO_2_, ZnO) are used as the switching medium in the MR devices, where the conducting filaments are created via the migration of the oxygen vacancies and/or the electrodes metal ions^17–21^. Few researchers are investigating the use of Graphene or Graphene Oxide (GO) as electrodes or switching material in MR; targeting neuromorphic behavior, due to the outstanding features of Graphene in terms of flexibility, low cost, adaptability, and being environmentally friendly^22–27^. It has been shown that deploying Graphene as electrodes in MR can increase the conductivity and therefore improve the performance of the device. Additionally, synthesizing MR with GO as switching material can assist the analog switching behavior which is considered an asset for neuromorphic applications.

To the best of our knowledge, this work is the first to report on a one directional planar flexible prGO-based MR, named NeuroMem, with analog switching behavior^22–26^. Being a one directional device, NeuroMem emulates the memorization behavior of humans, which is irreversible once new information is added. Forgetting information, after a certain duration, which is the natural action of the human brain can be mapped to the retention time of the MR. Moreover, the device exhibits an extremely analog behavior by demonstrating a continuous change in the device resistance depending on the applied voltage, compared to only six distinct states reported in the literature of Graphene-based memristors^22^. Furthermore, the prGO thin films in NeuroMem are fabricated from an aqueous solution of GO over a polymer flexible substrate by using standard microfabrication processes. This makes the process simple, cost effective, and suitable for mass production of the device. It also enables the fabrication of low-cost disposable flexible electronics. It is worth mentioning that NeuroMem is fabricated using low temperature process (below 70 °C) which enables compatibility with CMOS backend process for heterogeneous integration^28^. The planar nature of NeuroMem enables using multi-layer of the same technology. Moreover, it is considered a great asset for sensing applications as it provides more interaction volume^29^. Also, the planar structure is gaining great interest in the field of adaptable communication systems, for which the planar MR can be integrated easily to provide tunable feature to the associated communication component^30^. Furthermore, planar devices require less fabrication steps, especially if both electrodes are of the same material, which leads to lower cost and faster fabrication process. In this article, we have successfully utilized the analog behavior of NeuroMem device to fabricate MR crossbars to perform the computation needed by the inference engine in AI applications.

**Figure 1:**
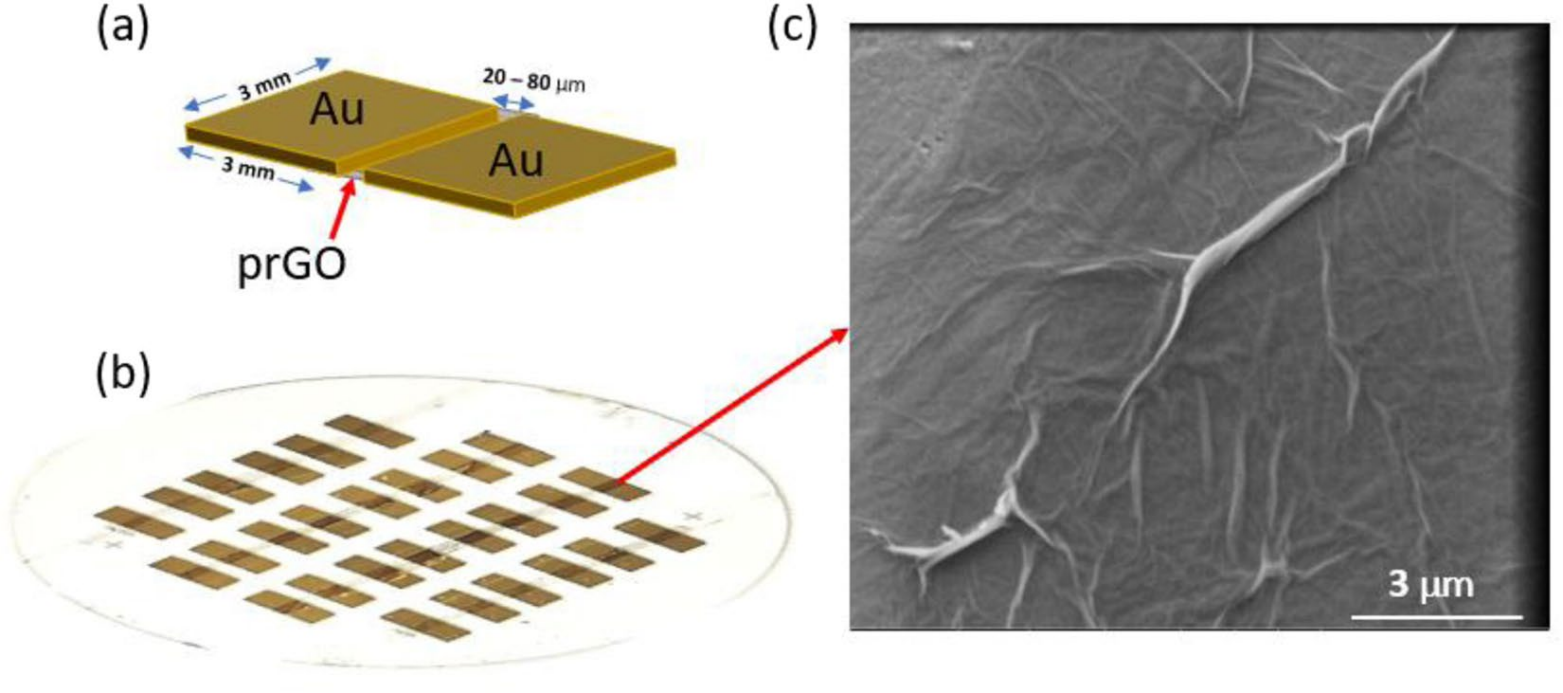
NeuroMem structure: (**a**) Device schematic to show the planar Au/prGO/Au structure (Drawing Software: Microsoft Powerpoint 2016). (**b**) A macrograph of the fabricated NeuroMem devices on COC substrate. (**c**) Scanning electron microphotograph of the deposited prGO layer, top view, under secondary electron mode (accelerating voltage, 5 kV; magnification, 24 000 ×; working distance, 10 mm).

As shown in Fig. 1(a), NeuroMem consists of one pair of first and second Au electrodes separated by a gap containing a thin film of prGO. The prGO film and metal electrodes rest on a COC substrate (Fig. 1(b)). Operating the device on a flexible substrate enables its integration in flexible electronics technology that allows building electronic circuits on flexible polymer substrates that can be bendable and/or stretchable. Flexible electronics have many applications including flexible sensors, flexible batteries, and flexible memory, which all are essential elements for wearable devices^31^. Thus, the flexible substrate of NeuroMem extends its potential to be integrated in smart wearable devices, where the flexible memory and Artificial Intelligence (AI) elements play vital role for such technology. The scanning electron microphotograph presented in Fig. 1(c) shows the morphology of the deposited prGO film at high magnification. The present work details the NeuroMem electrical characterization with a plausible elucidation of the switching phenomena associated with the device. Also, it presents the deployment of NeuroMem fabricated crossbars in artificial neural network (ANN) inference application. In addition, this article details the fabrication method of NeuroMem device.

## Results

### NeuroMem electrical characteristics

**Figure 2:**
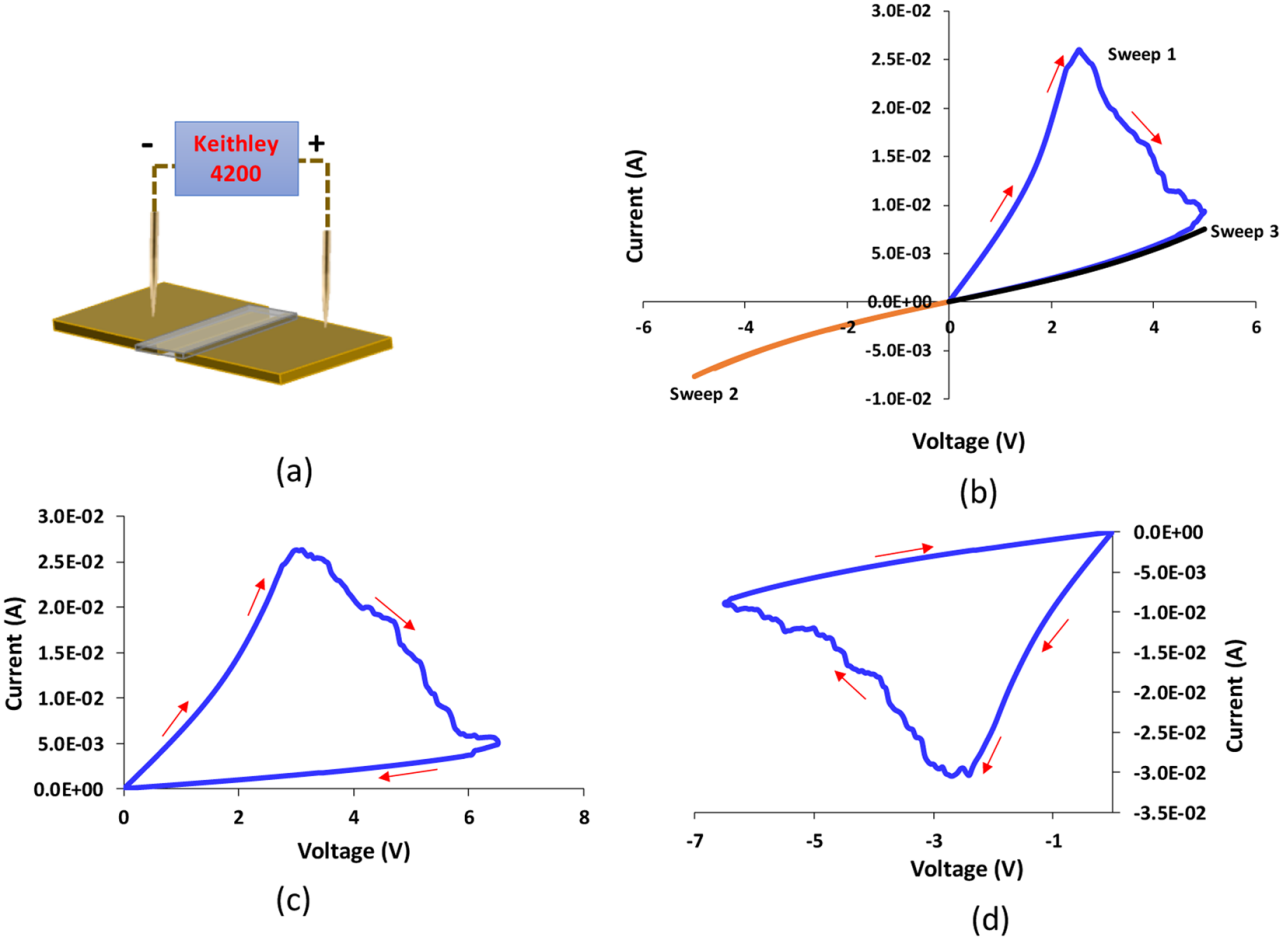
The data provided here is used to show the non-volatility of NeuroMem with the unipolar switching behavior. (**a**) A schematic to show the testing setup of NeuroMem. A chart illustrating the measured current across NeuroMem fresh devices with gap of 50 µm (Drawing Software: Microsoft Powerpoint 2016) (**b**) under the application of three consecutive voltage sweeps, (**c**) under the application of + 6 V voltage sweep, (**d**) under the application of − 6 V voltage sweep.

The data provided in Fig. 2 is used to show the non-volatility of NeuroMem with unipolar switching behavior. NeuroMem devices are characterized using Keithley 4200 SCS Parameter Analyzer (see Fig. 2(a)). Sweep mode is used to obtain Current-Voltage (*I-V*) characteristics for identical devices.

Fresh NeuroMem device is tested by consecutively applying positive, negative and then positive voltage sweeps. As shown in Fig. 2(b). The application of Sweep 1 puts the device in the OFF state. Under the application of Sweep 2 and 3, it is clear that the device holds the last written state and the initial ON state cannot be retrieved. This switching behavior mimics the memorization process of the brain as humans generally cannot unlearn information; however, it can be forgotten after a certain time. In MR devices this time can be compared to the retention time of the device. To confirm the unipolar switching behavior of NeuroMem, two fresh devices are characterized by applying +6.5 V and −6.5 V voltage sweeps, respectively. As presented in Fig. 2(c,d), NeuroMem preserves its behavior regardless of the applied polarity, rather it depends on the synergistic effect of the device current and the applied voltage.

**Figure 3:**
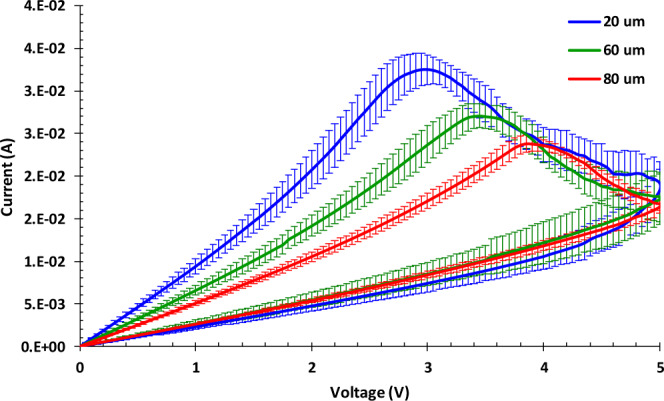
Chart illustrating the measured current across NeuroMem devices fabricated with gap of 20 µm, 60 µm, and 80 µm under the application of + 5 V voltage sweep. For each width gap, the illustrated data represents the average result obtained from 10 identical devices, with error bars for the measured current variation.

For NeuroMem, it is observed that the switching voltage reduces with a smaller gap size. This is confirmed by testing devices with different gaps (20 µm, 60 µm, and 80 µm) fabricated on the same wafer. As shown in Fig. 3, 10 identical devices are tested for each gap length to confirm the uniformity of the deposited prGO film and the reproducibility of the electrical characteristic of NueroMem. It can be observed that the switching voltage decreases when the gap decreases from 80 µm to 20 µm. This can be explained through the Joule heating taking place and contributing to the switching mechanism of the device. To elaborate more, the devices with shorter gap has lower resistance and consequently higher initial current. Thus, the power of heating needed to switch the device can be achieved at lower voltage.

When talking about MR devices switching, two terminologies can be distinguished; (i) the switching behavior and (ii) the switching mechanism. Switching behavior is related to the polarity of the applied voltage and how it does affect the state changing in memristors. Two switching behaviors can be observed in memristor devices; the bipolar and unipolar switching. In bipolar, the switching from OFF to ON, or reve rsely depends on the polarity of the voltage applied, while it relies on the voltage magnitude in unipolar devices. Based on that, NeuroMem can be considered as unipolar memristor as it can have similar *I-V* characteristics in both voltage polarities. As for the switching mechanisms, the most common mechanisms associated to memristor devices are (i) the valence change memory (VCM)^32^ and (ii) the electrochemical metallization (ECM)^33^ memory. These mechanisms depend on creating the conducting filaments through the migration of oxygen vacancies or the metal ions. This can be achieved by applying high electric field which in most of the available structures is achieved by fabricating nano-thick devices. In some cases, a thermochemical process is also provided in addition to VCM and ECM to explain the stoichiometric changes taking place in the switching layer, as a result of current-related thermal effects^21,34–36^.

**Figure 4:**
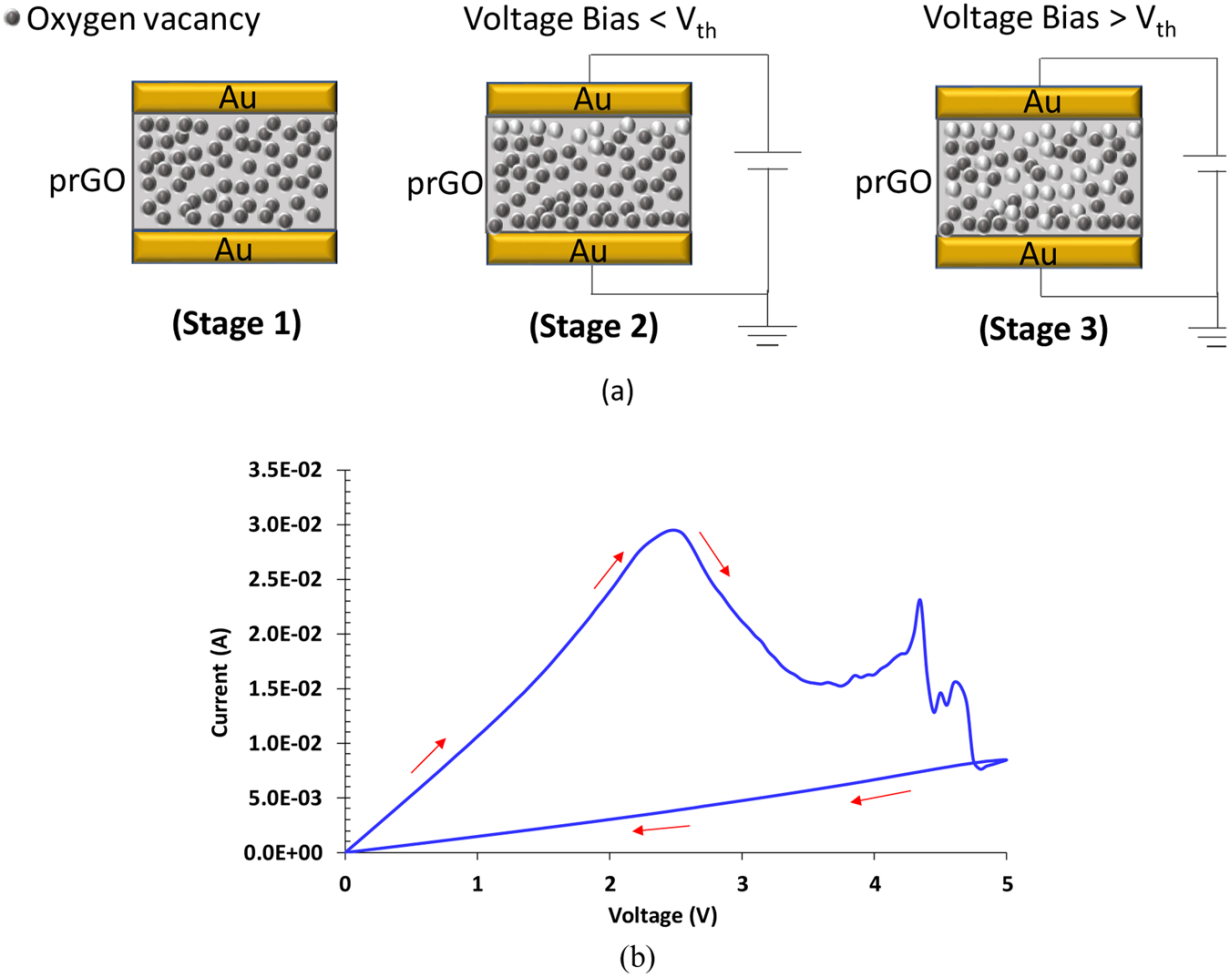
Switching Mechanism of NeuroMem. (**a**) Schematic representation of the switching phenomenon occurring in NeuroMem; (stage 1) pristine stage, (stage 2) after applying voltage of magnitude: Voltage Bias < Vth, (stage 3) after applying consecutive voltage sweeps of increasing magnitude: Voltage Bias > Vth. Dimensions are for illustration purposes. (**b**) A chart illustrating the measured I-V across NeuroMem fresh device with gap of 20 µm under vacuum (i.e. pressure = 5 × 10-5 Pa).

Here we give an insight into the possible switching phenomena associated with NeuroMem devices. As depicted in Fig. 4(a) (Stage 1), the active layer (prGO) is supposed to contain oxygen vacancies distributed among the deposited film. Following the results obtained in Figs. 2 and 3, the clockwise *I-V* loops recorded with NeuroMem gradually decline towards lower current values, which indicates a progressive buildup of internal resistance. Observing the device behavior when the applied voltage is less than the threshold voltage V_th_ (V_th_ depends on the gap width), a slight decrease in the resistance can be observed. This is represented in Fig. 4(a) (Stage 2), which is in agreement with the results reported in literature^37^. Ekis *et al*. proved that, in ambient atmosphere, partially reduced Graphene Oxide can be oxidized and reduced with positive and negative charge, respectively. The same authors reported that oxidation rate is smaller than reduction rate at similar magnitude bias, which explains the mild decrease in NeuroMem resistance when the applied voltage is less than V_th_. Upon increasing the voltage (i.e. V > V_th_), NeuroMem resistance starts to rise. A plausible explanation for this is the antifuse mechanism relying on the Joule heating effect^34^. According to this view, RESET operation occurs by heat-induced solid phase dissolution of oxygen species at very high current densities. Therefore, on the incident of writing a higher resistance state during a certain sweep, lowering the level of the current passing through the device, a higher threshold voltage is required in the following sweep to achieve the power of heating needed to perform the oxidation process. Therefore, NeuroMem resistance can be further increased and a new state is added into the device. To confirm the Joule heating effect in NeuroMem, the *I-V* characteristics is measured for a fresh device under vacuum (i.e. pressure = 5 × 10^−5^ Pa). As shown in Fig. 4(b), the device preserves its switching behavior which proves that Joule heating is the dominant force responsible of the switching taking place in NeuroMem devices.

### NeuroMem analog switching characteristics

**Figure 5:**
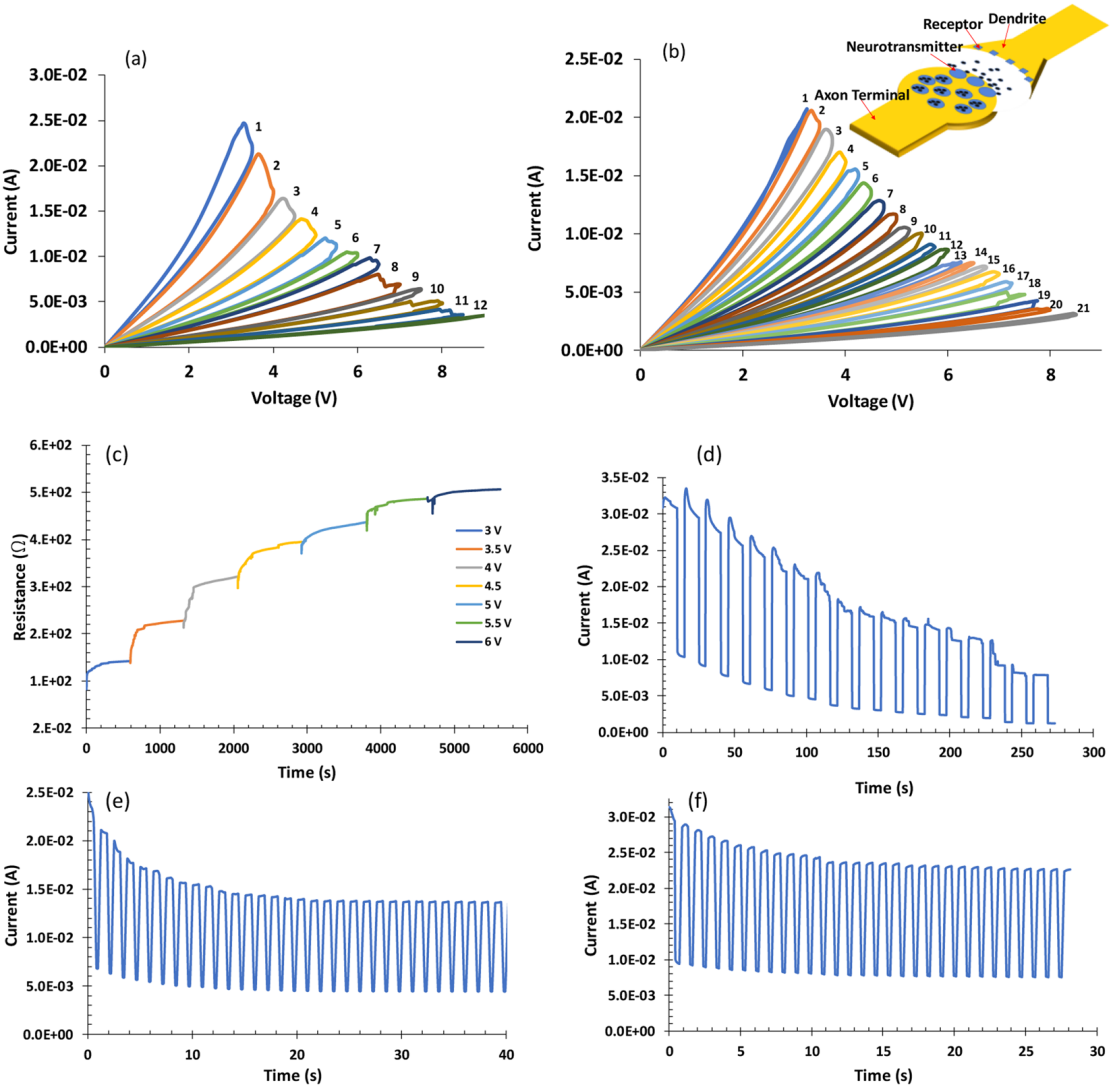
I-V traces and DC response of NeuroMem devices under the application of: (**a**) 12 voltage sweeps across fresh NeuroMem device, with a voltage step of 0.5 V. (**b**) 21 voltage sweeps across fresh NeuroMem device, with a voltage step of 0.25 V. (**c**) consecutive increasing writing voltage pulses from 3 V to 6 V with step of 0.5 V, gap of 50 µm (**d**) consecutive increasing writing voltage pulses from 2.5 V to 5.9 V with step of 0.2 V and 1 V reading pulses, the width of the writing and reading pulses is 10 s and 1 s, respectively, device gap is 50 µm (**e**) consecutive writing voltage pulses of 4 V and 1.5 V reading pulses, the width of the writing and reading pulses is 0.5 s and 0.2 s, respectively, device gap is 50 µm (**f**) consecutive writing voltage pulses of 4 V and 1.5 V reading pulses, the width of the writing and reading pulses is 0.5 s and 0.2 s, respectively, device gap is 80 µm.

To explore the analog behavior of the fabricated NeuroMem, a fresh device is characterized using consecutive voltage levels starting from 3 V, with an increment of 0.5 V in each sweep. The test is stopped once the device reaches the full OFF state. As shown in Fig. 5(a), a new state is written on the device when a new sweep with a higher voltage amplitude is applied. For each voltage the device has a maximum state drift that can be achieved. In such situation, the written state cannot be erased unless we apply a voltage sweep higher than the previous one.

To investigate the possibility of increasing the number of states generated in NeuroMem, a new device is characterized by applying successive voltage sweeps with an increment of 0.25 V in each sweep. Figure 5(b) shows that twenty-one distinct resistance states can be achieved by NeuroMem, compared to only six distinct states reported in the literature of Graphene-based memristors^22^. This can be further enhanced by tuning the voltage increment in each sweep. As it is presented in the following section, NeuroMem exhibits the ability to be tuned to any value, by careful optimization of the voltage pulse, within its R_ON_ and R_OFF_ range, which confirms the analog switching property of the device. As NeuroMem is one directional device, its endurance can be associated to its switching property. This means that the device can keep switching to new state as long as sufficient voltage is applied and the full OFF state (the maximum resistance the device can have) is not reached.

### NeuroMem potential application

The analog behavior exhibited by the fabricated NeuroMem makes it suitable for non-volatile multi-level cell (MLC) memories where higher density can be achieved compared to Flash, which is considered the best existing technology in the current market^36,38^. Furthermore, NeuroMem has much lower writing voltage when compared to 16 V needed for Flash cell^39^. The analog feature of NeuroMem, with the ability to mimic the memorization behavior of human brain, can be used to build artificial neural networks for neuromorphic computation which is important building block to enable AI^40^. The possibility of simulating the computation stages of the Convolutional Neural Network (CNN) deep learning approach through MR crossbars was investigated in^41^ and^42^. Although CNN structure was postulated by Fukushima in 1980^43^, it was difficult to be implemented due to its complicated training algorithms. This was overcome by the simplified algorithm proposed by LeCun *et al*. in the 1990s^44^. CNN structure consists mainly of the training (learning) stage and the inference (classification) stage^45^. The features extraction in CNN is performed by the convoltution layes and is considered the most computing intensive step. During the training stage, the weights of the CNN are calculated and the final values are used for the classification phase.

In this context, the non-volatility and highly analog behavior demonstrated by NeuroMem enable the device to act as an electronic synapse in CNN^45–48^. To eleborate, once the neural network is trained, NeuroMem crossbar can hold the synaptic weights in the clasification (inference) stage and perform dot-product operations. Depending on the target application, suitable mapping (conversion) algorithm is used to relate the final weights calculated during trainig stage to NeuroMem conductance range. As shown in Fig. 5(c–f), the width and amplitude of the applied voltage pulses control the number and the level of the conductances acheived in NeuroMem. The demonstration of utilizing NeuroMem crossbars for fully connected neural network to achieve classification of three-types of classes is detailed in the following section.

**Figure 6:**
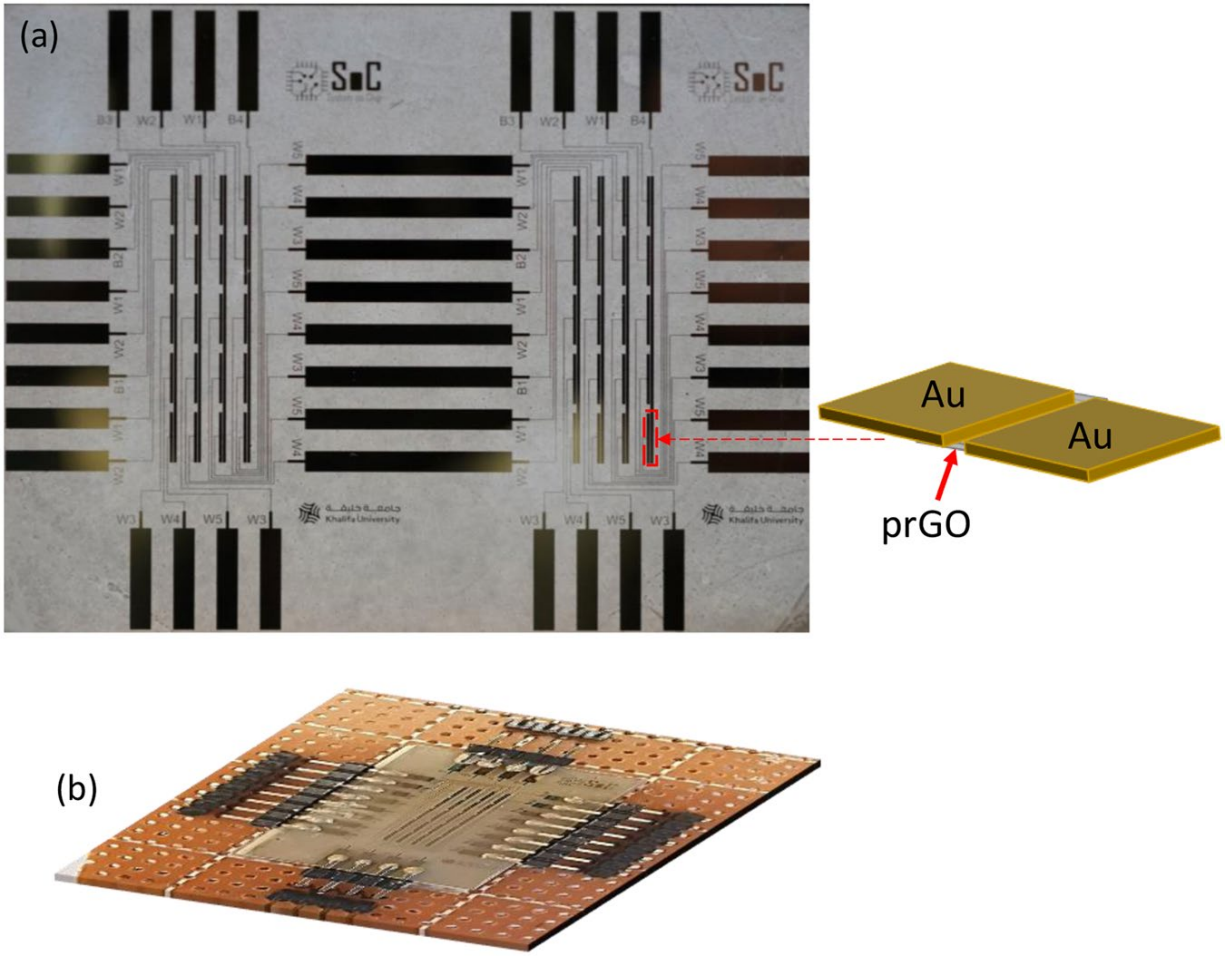
A macrograph of the fabricated NeuroMem-based ANN: (**a**) before, and (**b**) after cutting and conneting the crossbar to the electrical board. Figure inset shows the structure of one planar NeuroMem device pointed to one example location on the crossbar (Drawing Software: Microsoft Powerpoint 2016).

**Figure 7:**
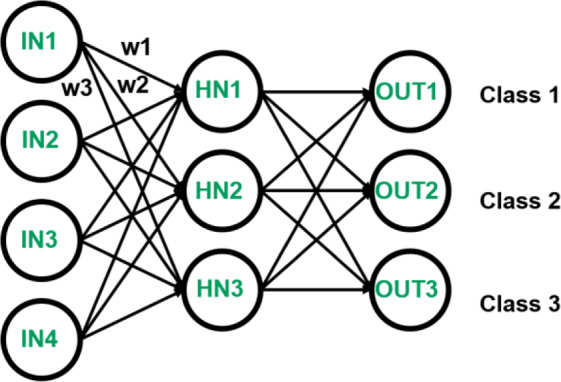
Graph representation of the perceptron with four input nodes, three hidden neurons, and three output classes. Not shown in the figure is the bias which has a value of 1 as an input and a weight connected to each hidden and output node.

**Figure 8:**
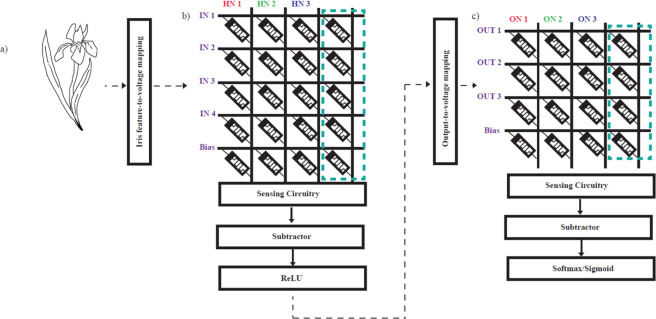
ANN architecture for Iris flower classification where (**a**) is the iris input that consists of the sepal and petal length and width in cm, (**b,c**) show the first and second layers of fabricated MR crossbars to accelerate the VMM of the classification task during inference phase. (HN represents the hidden neurons and ON represents the output neuron.).

Artificial Neural Network (ANN): Case Study. In order to demonstrate the potential of the NeuroMem device specially its analog memory characteristics which is important for many application, a NeuroMem-based ANN has been fabricated (shown in Fig. 6) and tested using Iris dataset^49^. The ANN consists of four input neurons, three hidden neurons and three output neurons as illustrated in Fig. 7 and used to c lassify the iris flower (Fig. 8(a)) based on its petal and sepal length and width into either Setosa, Versicolour, or Virginica classes. The main operation in any ANN heavily relies on the vector matrix multiplication (VMM) between the inputs and the network weights as in Eq. (1), which causes a bottleneck specially for resource-constrained edge computing devices. Such operations can be accelerated by utilizing the MR-based in-memory computing paradigm, where computations and storage are performed in the same memory cell^50^. The fabricated ANN consists of two layers: the first one produces intermediate results by multiplying and adding the input values with the hidden weights. The other layer is between the intermediate outputs that act as inputs to the output neurons.

1 $$\,[{\boldsymbol{OU}}{{\boldsymbol{T}}}_{1}\,{\boldsymbol{OU}}{{\boldsymbol{T}}}_{2}\,{\boldsymbol{OU}}{{\boldsymbol{T}}}_{3}]=[{\boldsymbol{I}}{{\boldsymbol{N}}}_{1}\,{\boldsymbol{I}}{{\boldsymbol{N}}}_{2}\,{\boldsymbol{I}}{{\boldsymbol{N}}}_{3}\,{\boldsymbol{I}}{{\boldsymbol{N}}}_{4}1]\cdot [\begin{array}{ccc}{{\boldsymbol{w}}}_{1} & {{\boldsymbol{w}}}_{2} & {{\boldsymbol{w}}}_{3}\\ {{\boldsymbol{w}}}_{4} & {{\boldsymbol{w}}}_{5} & {{\boldsymbol{w}}}_{6}\\ {{\boldsymbol{w}}}_{7} & {{\boldsymbol{w}}}_{8} & {{\boldsymbol{w}}}_{9}\\ {{\boldsymbol{w}}}_{10} & {{\boldsymbol{w}}}_{11} & {{\boldsymbol{w}}}_{12}\\ {{\boldsymbol{w}}}_{13} & {{\boldsymbol{w}}}_{14} & {{\boldsymbol{w}}}_{15}\end{array}]$$

ANN training is performed offline using MATLAB R2017a. Once the classification accuracy reached 96.67%, the training phase stops. After that, the fixed weights are mapped into MR conductance values, using the algorithm reported in^51^, that are within the R_ON_ and R_OFF_ range of NeuroMem. As NeuroMem has analog memory characteristics, the mapped conductance values are written to NeuroMem crossbars to perform the inference phase for Iris classification. The input, which is basically the sepal and petal length and width, is converted into normalized voltage amplitude to the terminals of the crossbar. Moreover, in order to mitigate the sneak path occuring through computation, a virtual ground must be connected to each bitline^50^.

ANN weights can have both positive and negative polarities. Negative representation of any arbitrary value of conductance is a challenge. One of the solutions proposed in the literature is to shift all weights by the minimum value as in^52^. The minimum and maximum resistance range exhibited by NeuroMem and used in the mapping algorithm is 110–900 Ω. At the end of the VMM operation, all the output results are shifted back by the following operation:

2 $$[{\boldsymbol{OU}}{{\boldsymbol{T}}}_{1}\,{\boldsymbol{OU}}{{\boldsymbol{T}}}_{2}\,{\boldsymbol{OU}}{{\boldsymbol{T}}}_{3}]=[{\boldsymbol{I}}{{\boldsymbol{N}}}_{1}\,{\boldsymbol{I}}{{\boldsymbol{N}}}_{2}\,{\boldsymbol{I}}{{\boldsymbol{N}}}_{3}\,{\boldsymbol{I}}{{\boldsymbol{N}}}_{4}1][\begin{array}{ccc}{{\boldsymbol{w}}}_{1}+|{{\boldsymbol{w}}}_{{\boldsymbol{\min }}}| & {{\boldsymbol{w}}}_{2}+|{{\boldsymbol{w}}}_{{\boldsymbol{\min }}}| & {{\boldsymbol{w}}}_{3}+|{{\boldsymbol{w}}}_{{\boldsymbol{\min }}}|\\ {{\boldsymbol{w}}}_{4}+|{{\boldsymbol{w}}}_{{\boldsymbol{\min }}}| & {{\boldsymbol{w}}}_{5}+|{{\boldsymbol{w}}}_{{\boldsymbol{\min }}}| & {{\boldsymbol{w}}}_{6}+|{{\boldsymbol{w}}}_{{\boldsymbol{\min }}}|\\ {{\boldsymbol{w}}}_{7}+|{{\boldsymbol{w}}}_{{\boldsymbol{\min }}}| & {{\boldsymbol{w}}}_{8}+|{{\boldsymbol{w}}}_{{\boldsymbol{\min }}}| & {{\boldsymbol{w}}}_{9}+|{{\boldsymbol{w}}}_{{\boldsymbol{\min }}}|\\ {{\boldsymbol{w}}}_{10}+|{{\boldsymbol{w}}}_{{\boldsymbol{\min }}}| & {{\boldsymbol{w}}}_{11}+|{{\boldsymbol{w}}}_{{\boldsymbol{\min }}}| & {{\boldsymbol{w}}}_{12}+|{{\boldsymbol{w}}}_{{\boldsymbol{\min }}}|\\ {{\boldsymbol{w}}}_{13}+|{{\boldsymbol{w}}}_{{\boldsymbol{\min }}}| & {{\boldsymbol{w}}}_{14}+|{{\boldsymbol{w}}}_{{\boldsymbol{\min }}}| & {{\boldsymbol{w}}}_{15}+|{{\boldsymbol{w}}}_{{\boldsymbol{\min }}}|\end{array}]\,$$

To get the same output results as in Eq. (1), the contribution of $|{{w}}_{{\min }}|$is removed by

3 $$[\begin{array}{ccc}{\boldsymbol{OU}}{{\boldsymbol{T}}}_{1}-(|{{\boldsymbol{w}}}_{{\boldsymbol{\min }}}|\ast \mathop{\sum }\limits_{{\boldsymbol{i}}=1}^{5}{\boldsymbol{I}}{{\boldsymbol{N}}}_{{\boldsymbol{i}}}) & {\boldsymbol{OU}}{{\boldsymbol{T}}}_{2}-(|{{\boldsymbol{w}}}_{{\boldsymbol{\min }}}|\ast \mathop{\sum }\limits_{{\boldsymbol{i}}=1}^{5}{\boldsymbol{I}}{{\boldsymbol{N}}}_{{\boldsymbol{i}}}) & {\boldsymbol{OU}}{{\boldsymbol{T}}}_{3}-(|{{\boldsymbol{w}}}_{{\boldsymbol{\min }}}|\ast \mathop{\sum }\limits_{{\boldsymbol{i}}=1}^{5}{\boldsymbol{I}}{{\boldsymbol{N}}}_{{\boldsymbol{i}}})\end{array}]$$

where $OU{T}_{i}$ is the output calculated from multiplying and adding the inputs by the weights. The biases are represented by ${w}_{13}-{w}_{15}$ and multiplied by 1.

In MR crossbar hardware implementation presented in Fig. 8(b,c), this operation can be translated into adding a fourth column of MR devices to hold the minimum weight value, hence a 5 × 4 and a 4 × 4 crossbar sizes are fabricated. Then each output is subtracted from the fourth column and after that it is passed through the activation function ReLU. The output results serve as input to the output layer, as illustrated in Fig. 8(c). Moreover, other VMM operations take place, then the results are shifted back and passed through the sigmoid function to produce the final output which is the class of the input sample. The classification accuracy with the ideal mapped conductance values is 96.67%, compared to 93.33% achieved using the fabricated NeuroMem-based ANN. To further confirm the high retention of NeuroMem, the resistance states of the devices fabricated in the two crossbars used to demonstrate the inference application were measured two months after writing the weights values on them. The devices showed to hold and remember the last states written to them.

A critical parameter in analog computation is the accuracy of analog tuning of the MR conductance value. Tables 1 and 2 demonstrate the absolute percentage of the deviation of the physical programmed conductance from the ideal shifted weight value obtained from MATLAB. For example, 0% in Table 1 HN1(1) means we were able to write the ideal value in conductance with very high accuracy. While a 9% as in Table 2 Constant Term(1), indicates a +/- deviation from the true value. It was observed that classification accuracy is more sensitive to programming errors in high conductance values since they allow higher currents to contribute to the multiply and add output results per neuron (column). It is worth mentioning that the conductance writing errors shown in the tables can be further reduced by careful optimization and tuning of the writing voltage pulses used to change the conductivity of the NeuroMem devices.

**Table 1:** Hidden Layer (HN) (Fig. 8b) Error Matrix showing the Absolute Deviation of the Physical Programmed Conductance Values and the Ideal Mapped Neural Network Values per Device.

HN1	HN2	HN3	Constant Term
0%	0%	9%	1%
6%	1%	5%	0%
2%	8%	1%	8%
13%	1%	0%	2%
5%	5%	6%	3%

**Table 2:** Output Layer (ON) (Fig. 8c) Error Matrix showing the Absolute Deviation of the Physical Programmed Conductance Values and the Ideal Mapped Neural Network Values per Device.

ON1	ON2	ON3	Constant Term
10%	3%	2%	9%
11%	1%	5%	4%
0%	1%	0%	2%
0%	7%	1%	3%

## Conclusion

In conclusion, this work presented novel planar MR device, NeuroMem, that consists of Au/prGO/Au. NeuroMem exhibited unique behavior with over twenty different resistance states being generated with the application of consecutive voltage sweeps. After each new writing process, NeuroMem preserved its last state and required a higher voltage amplitude to move to the next level. The use of symmetrical electrodes aided the unipolar switching behavior of the device. Thus, operating NeuroMem did not depend on the voltage polarity, instead, the writing operation depended on the applied voltage magnitude and its previous state. Moreover, the prGO layer deposited between the metal electrodes exceled the analog switching characteristic of NeuroMem with its irreversibility feature. In this work, NeuroMem-based crossbars were fabricated and shown to act as electronic synapse in ANN. The devices were successfully utilized to perform inference (classification) in fully connected network. NeuroMem is considered a progressive step towards the deployment of flexible electronics in AI systems to perform efficient computing especially at the edge devices^53^. The main aim of this work is to report on a novel analog RRAM device that can be utilized to perform inference for AI applications. After this concept has been proved and demonstrated, the next step is to scale and optimize the device/crossbar dimensions to be compatible with real world application. This can be achieved by diminishing the device geometry. Moreover, the stacked structure can be investigated and utilized if more efficient area can be achieved.

## Experimental Section

**Figure 9:**
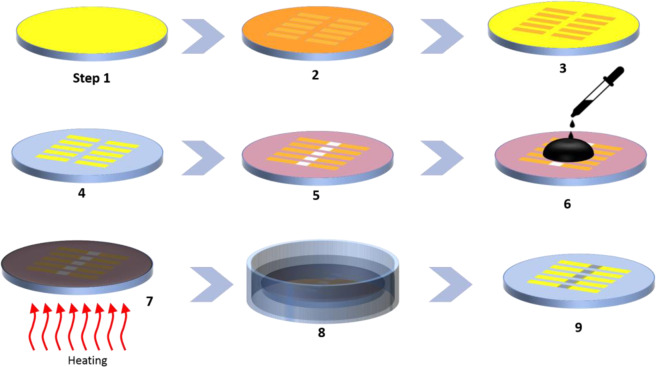
The flow chart schematic diagram of the fabrication process for NeuroMem devices. (Drawing Software: Microsoft Powerpoint 2016).

NueroMem is fabricated on COC substrate using standard microfabrication techniques. The process layout of the fabrication process is shown in Fig. 9. A clean COC wafer is sonicated in Isopropanol and distilled water baths, dehydrated using compressed Nitrogen, baked, and then a thin layer of gold is deposited and patterned by wet chemical etching technique (steps 1–4). To achieve this, a sputter is used to initially depositing the gold film on the COC wafer and then a thin layer of 1813 positive photoresist is deposited on top of the gold film using a spin coater. The photoresist layer is patterned using a photolithography system (Dilase KLOE 650) and then developed using a proper developer (steps 2 and 3). The exposed gold film is then etched away using gold etchant leaving the gold regions of the film that are masked by the photoresist layer (step 4). Then, the photoresist layer is stripped-off using acetone. After that, a layer of GO is deposited and patterned on top of the gold film using plasma-enhanced liftoff approach^54^. A photoresist layer is initially patterned on top of the gold patterned layer (step 5), the substrate is then treated with plasma and a thin film of GO is deposited using a spin coater (step 6). The substrate is then baked on a hot plate (step 7), and then the photoresist layer is removed using liftoff process. The final step in the fabrication process is the reduction of the patterned GO f ilm into prGO. This is achieved chemically by immersing the COC substrate in Hydroiodic acid (step 8). The fabricated NeuroMem devices are schematically shown in step 9 of Fig. 9.
